# A new approach to comparing the demands of small-sided games and soccer matches

**DOI:** 10.5114/biolsport.2024.132989

**Published:** 2023-12-20

**Authors:** Mauro Mandorino, Antonio Tessitore, Sebastien Coustou, Andrea Riboli, Mathieu Lacome

**Affiliations:** 1 Performance and Analytics Department, Parma Calcio 1913, 43121 Parma, Italy; 2 Department of Movement, Human and Health Sciences, University of Rome “Foro Italico”, Piazza L. de Bosis 6, 00135 Rome, Italy; 3 MilanLab Research Department, AC Milan S.p.a., Milan, Italy; 4 Department of Biomedical Sciences for Health, Università degli Studi di Milano, Milan, Italy; 5 French Institute of Sport (INSEP), Research Department, Laboratory Sport, Expertise and 11 Performance (EA 7370), Paris, France

**Keywords:** Euclidean distance, Performance, External load, Overload, Similarity

## Abstract

To improve soccer performance, coaches should be able to replicate the match’s physical efforts during the training sessions. For this goal, small-sided games (SSGs) are widely used. The main purpose of the current study was to develop similarity and overload scores to quantify the degree of similarity and the extent to which the SSG was able to replicate match intensity. GPSs were employed to collect external load and were grouped in three vectors (kinematic, metabolic, and mechanical). Euclidean distance was used to calculate the distance between training and match vectors, which was subsequently converted into a similarity score. The average of the pairwise difference between vectors was used to develop the overload scores. Three similarity (Sim_kin_, Sim_met_, Sim_mec_) and three overload scores (OVER_kin_, OVER_met_, OVER_mec_) were defined for kinematic, metabolic, and mechanical vectors. Sim_met_ and OVER_met_ were excluded from further analysis, showing a very large correlation (*r* > 0.7, *p* < 0.01) with Sim_kin_ and OVER_kin_. The scores were subsequently analysed considering teams’ level (First team vs. U19 team) and SSGs’ characteristics in the various playing roles. The independent-sample *t*-test showed (p < 0.01) that the First team presented greater Sim_kin_ (*d* = 0.91), OVER_kin_ (*d* = 0.47), and OVER_mec_ (*d* = 0.35) scores. Moreover, a generalized linear mixed model (GLMM) was employed to evaluate differences according to SSG characteristics. The results suggest that a specific SSG format could lead to different similarity and overload scores according to the playing position. This process could simplify data interpretation and categorize SSGs based on their scores.

## INTRODUCTION

Success in soccer is determined by the interaction of several factors, among which technical skills, physical capabilities, and tactical knowledge represent the most important performance elements [1]. Following the principle that “you should train as you play” [2], coaches should aim to develop training methods capable of replicating match intensity demands. Indeed, in the theory of training, the principle of specificity suggests that performance increases when training is able to replicate the physiological demands and movement patterns occurring during competitive matches [3]. For this reason, small-sided games (SSGs) became a very popular training method due to their ability to concomitantly train technical, tactical, and physical aspects by manipulating different variables such as pitch size, the number of players, recovery periods, the presence of goal-keepers, and playing rules [4–10].

In general, it has been reported that a greater number of players and a larger pitch area lead to higher physical demand in SSGs [11, 12]. Clemente et al. [13] observed that the 4 v 4 format induced players’ greater distance coverage and speed compared to the 2 v 2 format. Similarly, Lacome et al. [14] reported a higher overall running intensity (total distance and high-speed running) during 10 v 10 compared with 8 v 8, 6 v 6, and 4 v 4 formats. Regarding the pitch area, several studies reported that, by increasing the area per player, it was possible to induce higher locomotor demands for total distance, high-speed, and very high-speed distance [15, 16]. However, by reducing the size of the pitch, Gaudino et al. [17] registered a more significant number of moderate accelerations and decelerations with a higher number of changes in velocity. For this reason, with appropriate SSG formats, coaches and physical trainers can demand specific movements patterns and elicit players’ responses according to pre-defined targets of the training session (e.g., strength, endurance, speed).

Global positioning systems (GPSs) and radio-frequency local positioning systems (LPSs) are typically used to quantify the locomotor patterns and the intensity achieved in SSGs and to compare them with the match demands. These systems can provide several work-load metrics, with some studies reporting more than 80 parameters [18]. All these variables are used to quantify the locomotor patterns. They are generally classified into kinematic (i.e., the overall movement performed by players at different running speeds), metabolic (i.e., estimated energy cost of activity during training and matches) [19], and mechanical (i.e., the overall load placed on the body during accelerations and decelerations) [20]. Despite the large amount of data and information provided by GPSs, many studies are limited to using a few variables to compare SSGs and matches. For example, Beenham et al. [21] analysed differences between SSGs and match play using only player load. For the same purpose, Dalen et al. [22] investigated differences only in acceleration and high-intensity activities. Although using a few variables could ease the interpretation, the metrics selected could not capture a significant proportion of information provided by multiple load variables [23]. Conversely, Gómez-Carmona et al. [24] used a pairwise comparison to analyse differences between SSGs and official matches regarding 31 different variables. However, in this case, we can identify the following limitations: (1) risk of “data overload” [23]; (2) increasing the number of variables also increases the difficulty in interpretation; (3) a larger number of variables makes data visualization and communication with the coach difficult; (4) complexity in the staff decision-making processes.

To avoid losing important information from multiple variables, while attempting to overcome the “data overload” problem, we want to present a new approach to provide a practical and easy quantification of training efforts compared to a match. To quantify the similarity between SSGs and soccer match locomotor demands (kinematic, metabolic, mechanical), Euclidean distance calculation was employed. Euclidean distance is a very simple similarity metric which reflect the distance between the vectors being analysed. In the current study, we considered two different vectors: the SSG vector and the match vector. If the Euclidean distance is very small, then the values in each vector are very similar, and this suggests a high similarity between the two vectors. Therefore, the Euclidean distance offers several advantages within the context of the current study: (1) it allows multiple variables to be treated as vectors (GPS metrics), thereby avoiding the risk of “data overload”; (2) it is easy to implement; (3) it is straightforward to interpret. However, also knowing the similarity between training and match play is not enough. Indeed, according to the principle of training, it is necessary to create a progressive overload to elicit adaptation processes in the physiological systems [25]. To achieve this goal, the average of the pair-wise difference between the two vectors was calculated.

Therefore, the main purpose of the current study was to introduce a similarity and overload score to compare SSG and match demands based on external load variables. Particularly, for each SSG, three similarity scores and three overload scores were calculated for kinematic, metabolic, and mechanical variables. In addition, we evaluated whether and how these similarity and overload scores changed according to the SSGs’ characteristics (i.e., area per player, type of drills) in the different playing roles.

## MATERIALS AND METHODS

### Participants

A total of fifty-one elite soccer players were involved in the present study: twenty-six from the First team (age: 24.5 ± 5.5; body mass: 80.9 ± 6.9; height: 184.9 ± 6.1) and twenty-five from the U19 team (age: 17.4 ± 0.9; body mass: 73.9 ± 6.6; height: 181.4 ± 6.5). All participants were classified according to their playing position: defenders (n = 20), midfielders (n = 17), and forwards (n = 14). The goalkeepers were excluded from the data collection. Data were obtained from daily monitoring of the routine over the course of the competitive season. Therefore, the usual appropriate ethics committee clearance was not required [26]. Nevertheless, to guarantee team and player confidentiality, all data were anonymized before analysis, and the study was conducted following the Code of Ethics of the World Medical Association (Declaration of Helsinki).

### Experimental Approach

The study was conducted during the 2021/22 soccer season, involving players from a First team and a U19 team competing in the Italian Serie B and Primavera 2 championships, respectively, and belonging to the same Italian professional soccer club. The two teams trained five days per week and competed once a week. In all, 128 training sessions were monitored, with a total of 3290 individual observations. Only players free from injury involved in the full training schedules were considered. In the end, 147 different SSGs were selected for further analysis. All SSGs were performed under the supervision and motivation of the coaching staff to keep the work rate high. The different SSGs were classified based on the type of drill:

– Game Simulations: games performed with two goals (regular size) and the presence of goalkeepers.
– Possession Games: possession drills performed without goalkeepers.
– Tactical Games: games performed with two goals (regular size) and specific tactical rules (e.g., presence of time constraints, pressing rules, presence of limitations in the playing space).

The area per player (ApP) [27, 28]:

– Small Games: ApP ≤ 100 m^2^
– Medium Games: 100 m^2^ < ApP ≤ 200 m^2^
– Large Games: ApP > 200 m^2^

Following the purpose of the study, the players’ efforts during SSGs were subsequently compared with players’ efforts elicited during matches. Twenty-eight different matches were monitored for the First team, and twenty-two for the U19 team.

### Data Collection

The players’ external training/match load was collected using a 10-Hz GPS (WIMU PRO; RealTrack Systems SL) with an integrated 100 Hz tri-axial accelerometer, gyroscope, and magnetometer. Data were subsequently analysed using the system-specific software (WIMU Software; RealTrack Systems SL). The GPS system showed good accuracy for measures of running speed, acceleration, and deceleration in previous studies [29, 30]. The GPS devices were placed between the players’ scapulae through a tight vest. Among the numerous GPS variables, 19 different metrics were extracted and classified as kinematic, metabolic, and mechanical [20]. All the variables that constitute the vectors are presented in Table 1.

**TABLE 1:** Kinematic, metabolic, and mechanical variables selected for similarity and overload score estimation.

KINEMATIC	METABOLIC	MECHANICAL
Total Distance	Energy Expenditure	Number of accelerations above 2.5 m/s^2^
Distance above 7.2 km/h	Average Metabolic Power	Number of accelerations above 3.5 m/s^2^
Distance above 14.4 km/h	Distance covered from 5 to 10 W	Number of accelerations above 4.5 m/s^2^
Distance above 19.8 km/h	Distance covered from 10 to 20 W	Number of decelerations below -2.5 m/s^2^
Distance above 25.2 km/h	Distance covered from 20 to 35 W	Number of decelerations below -3.5 m/s^2^
Max Speed	Distance covered from 35 to 55 W	Number of decelerations below -4.5 m/s^2^
	Distance covered above 55 W	

### Similarity Score and Overload Score Estimation

As previously mentioned, we grouped GPS variables into three classes we identified as our three vectors. The different GPS metrics were normalized according to training duration to allow the comparison with matches. Therefore, each SSG was characterized by three different vectors that were compared with the benchmark match vectors. The three benchmark match vectors (kinematic, metabolic, mechanical) were calculated for each player as the average (µ_p_) and standard deviation (σ_p_) of the different GPS variables. The vectors were calculated for the 90-minute time window of the match. If 90-minute data were unavailable, calculations were made based on the time played. In this case, only players’ matches with a time of play over 45 minutes were considered. Also in this case, data were expressed per minute played. The benchmark match vectors and SSG vectors were generated for the various subjects, thereby enabling a comparative analysis of each player with oneself.

The average and standard deviation calculated for the matches were used to centre and scale training data using the following formula:

$$Ci=\frac{\left(Ti-\mu i\right)}{\sigma i}$$

C*i* = centred and scaled training data

T*i* = training data

µ_i_ = average match data

σ*i* = standard deviation match data

Then, the Euclidean distance (= L2-Norm) of each drill vector was calculated:

$$Di=∥Ci{∥}_{2}$$

At this stage, the distance value obtained was converted to a similarity score using the following formula:

$$Simk=\frac{1}{1+\frac{Di}{Ni}}$$

Where Ni represents the number of variables that constitute the vector. Therefore, for each drill and each player, we identified three different similarity scores for the kinematic vector (Sim_kin_), metabolic vector (Sim_met_), and mechanical vector (Sim_mec_). The similarity score could range from 0, which means “inability to replicate match demands”, to 1, which stands for the “maximum ability to replicate match demands”.

In addition, to determine whether the training (SSG) was globally more demanding or less demanding than the match, the average of the pairwise difference between the training vectors and benchmark match vectors was calculated:

Δ0=Avg(Ci)

Also in this case, for each drill and each player, we identified three different overload scores for the kinematic vector (OVER_kin_), metabolic vector (OVER_met_), and mechanical vector (OVER_mec_). A negative value indicates a lower overall intensity of the SSG compared with the match; conversely, a positive value indicates a higher overall intensity.

Therefore, the general demand of each drill was quantified according to the ability to replicate the match effort (similarity score) and the ability to overload players regarding match locomotor requests (overload score).

**FIG. 1:**
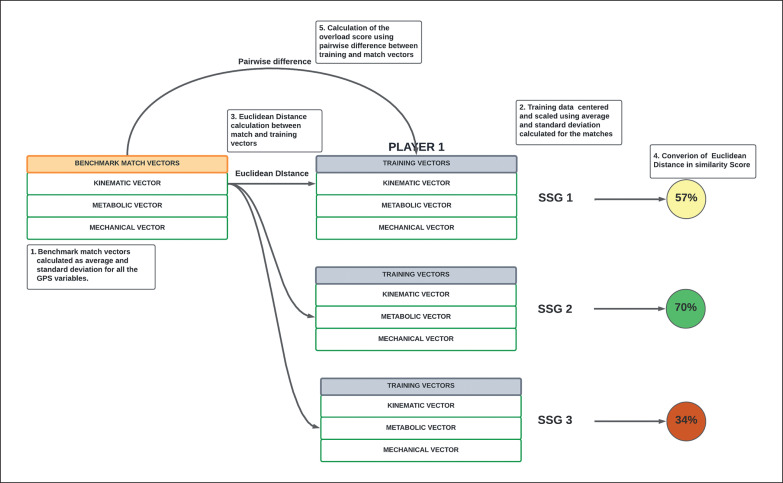
Summary of the steps to calculate similarity score and overload score. Note: SSG = small-sided game

The different steps followed to calculate the two scores are summarized in Figure 1.

### Statistical Analyses

Pearson’s correlation coefficient was used to evaluate the relationship between the different similarity scores and overload scores calculated. Correlation coefficient magnitudes were rated as trivial (*r* < 0.1), small (0.1 < *r* < 0.3), moderate (0.3 < *r* < 0.5), large (0.5 < *r* < 0.7), very large (0.7 < *r* < 0.9), and nearly perfect (*r* > 0.9) and perfect (*r* = 1.0) [31]. An independent-sample *t*-test was performed to test differences in the similarity scores and overload scores between the two different teams (First team vs U19 team). Cohen’s effect size was calculated and the magnitudes of the effect were interpreted according to the Hopkins criteria [32]: < 0.2 (*trivial*), 0.20–0.59 (*small*), 0.60–1.19 (*moderate*), 1.20–1.99 (*large*), 2.00–3.99 (*very large*), ≥ 4.00 (*nearly perfect*). To account for differences between similarity scores and overload scores with SSG characteristics, a generalized linear mixed model (GLMM) was employed. The characteristics of SSGs (i.e., type of the drill, ApP) were inserted into the model as fixed factors. The players’ identity was inserted into the model as a random effect to take into account the repeated measurements. GLMM was employed to understand the relationship between all the possible two-way interactions, and the similarity and overload scores were identified as the dependent variables. The dataset was split into three subsets according to players’ positions. Inside each subset, six GLMM were fitted, three for the similarity scores and three for the overload scores. Each standardized regression coefficient (*β*) was used to quantify the effect size of the individual predictors and ascertain which interaction was the most important in explaining the variation in the dependent variable [33, 34]. Data are presented as mean (± SD) and 95% confidence intervals. The significance level was set at p < 0.05. The software used for the statistical analysis of the data was IBM’s SPSS Statistics version 27 (SPSS, Inc. Chicago, Illinois IBM Corp., Armonk, NY).

## RESULTS

**FIG. 2:**
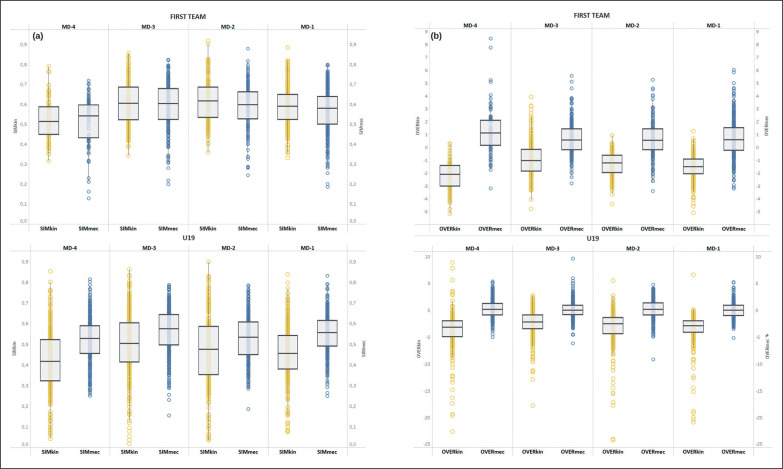
Weekly distribution of similarity (a) and overload (b) scores for the First team and U19 team. Note: MD = Match Day Simkin = similarity score for the kinematic vector OVERkin = Overload score for the kinematic vector Simmec = Similarity score for the mechanical vector OVERmec = Overload score for the mechanical vector

After correlation analysis, Sim_kin_ score showed a very large correlation (r = 0.892, p < 0.01) with Sim_met_ score, while Over_kin_ score showed a very large correlation (r = 0.708, p < 0.01) with Over_met_. For this reason, showing the same behaviour in relation to the SSGs, Sim_met_ score and Over_met_ score were excluded from further analysis. The 147 different SSGs were reported in the supplementary material (see Table S1) with their respective average values of similarity (Sim_kin_, Sim_mec_) and overload scores (OVER_kin_, OVER_mec_). The weekly distribution of similarity and overload scores for the two different teams is presented in Figure 2. The days of the week were classified according to the days preceding a match (MD-4; MD-3; MD-2; MD-1).

The independent-sample *t*-test revealed significantly higher similarity scores and overload scores (*p* < 0.01) in the First team compared to the U19 team except for Sim_mec_, for which no significant differences were found. A moderate effect was found for the Sim_kin_ score (*d* = 0.91), while a small effect was found for OVER_kin_ (*d* = 0.47) and OVER_mec_ (*d* = 0.35). Descriptive statistics for both groups are displayed in Table 2. Regarding the GLMM analysis, the results of the two-way interactions for the three different playing roles are summarized in Table 3. The two-way interactions (type of drill, ApP) produced eight different combinations (e.g., game simulations – small games, possession games – medium games) that were compared with the drill “tactical games – medium games” identified as the reference category in all the models. Tactical games – small games format was not included in the analysis as it was never recorded during the training sessions.

**TABLE 2:** Differences of similarity and overload scores between First team and U19 team after independent-sample t-test.

	Team	Mean	SD	p-value	Effect size (*d*)
Sim_kin_	U19 team	0.460	0.158	**0.001**	**0.91**
	First team	0.586	0.114		

OVER_kin_	U19 team	-3.213	5.146	**0.001**	**0.47**
	First team	-1.451	1.239		

Sim_mec_	U19 team	0.534	0.116	0.562	
	First team	0.567	0.119		

OVER_mec_	U19 team	0.066	1.929	**0.001**	**0.35**
	First team	0.675	1.510		

Sim_kin_ = similarity score for the kinematic vector; OVER_kin_ = Overload score for the kinematic vector; Sim_mec_ = Similarity score for the mechanical vector; OVER_mec_ = Overload score for the mechanical vector.

**TABLE 3:** Analysis of differences in similarity and overload scores according to SSGs characteristics and playing roles.

SIMILARITY SCORE (KINEMATIC VECTOR)											OVERLOAD SCORE (KINEMATIC VECTOR)								

DEFENDERS					MIDFIELDERS			FORWARDS			DEFENDERS			MIDFIELDERS			FORWARDS		

Type of the drill	ApP	β	95% CI	*p*-value	β	95% CI	*p*-value	β	95% CI	*p*-value	β	95% CI	*p*-value	β	95% CI	*p*-value	β	95% CI	*p*-value
(Intercept)		0.365	[0.252 0.478]	*0.001*	0.457	[0.395 0.518]	*0.001*	0.430	[0.326 0.492]	*0.001*	-5.945	[-9.465 -2.426]	*0.001*	-3.220	[-4.033 -2.408]	*0.001*	-3.772	[-5.117 -2.426]	*0.001*
GS	LG	**0.189**	[0.060 0.318]	**0.001**	**0.130**	[0.062 0.198]	**0.001**	**0.174**	[0.059 0.228]	**0.003**	**5.470**	[1.517 9.423]	**0.007**	2.042	[1.143 2.940]	**0.001**	2.482	[0.988 3.976]	**0.001**
	MG	0.148	[0.019 0.277]	**0.024**	0.078	[0.010 0.145]	**0.024**	0.129	[0.0144 0.216]	**0.028**	4.027	[0.103 7.951]	**0.044**	1.072	[0.175 1.968]	**0.019**	1.727	[0.240 3.214]	**0.023**
	SG	0.046	[-0.090 0.184]	0.501	-0.024	[-0.102 0.052]	0.530	0.032	[-0.09 10.156]	0.603	1.607	[-2.779 5.994]	0.471	-0.139	[-0.164 0.886]	0.789	0.596	[-1.014 2.208]	0.464

PG	LG	-0.009	[-0.165 0.146]	0.905	-0.045	[-0.132 0.041]	0.307	0.008	[-0.141 0.159]	0.907	1.995	[-2.866 6.857]	0.419	**3.688**	[2.538 4.837]	**0.001**	**2.165**	[0.214 4.115]	**0.030**
	MG	0.110	[-0.022 0.243]	0.104	0.052	[-0.018 0.124]	0.145	0.071	[-0.04 6.189]	0.234	2.698	[-1.446 6.843]	0.200	0.912	[0.162 1.765]	**0.045**	1.400	[0.117 2.933]	**0.041**
	SG	0.002	[-0.128 0.134]	0.966	-0.076	[-0.147 -0.004]	**0.036**	-0.013	[-0.132 0.105]	0.825	-1.143	[-4.218 3.931]	0.945	-0.929	[-1.871 -0.171]	**0.042**	-0.074	[-1.618 1.469]	0.924

TG	LG	0.070	[-0.073 0.215]	0.334	0.0247	[-0.048 0.097]	0.503	0.064	[-0.063 0.192]	0.318	1.513	[-2.830 5.858]	0.492	0.473	[-0.493 1.440]	0.334	0.877	[-0.786 2.541]	0.297

	MG	0a			0a			0a			0a			0a			0a		


**SIMILARITY SCORE (MECHANICAL VECTOR)**											**OVERLOAD SCORE (MECHANICAL VECTOR)**								

**DEFENDERS**					**MIDFIELDERS**			**FORWARDS**			**DEFENDERS**			**MIDFIELDERS**			**FORWARDS**		

**Type of the drill**	**ApP**	**β**	**95% CI**	***p*-value**	**β**	**95% CI**	***p*-value**	**β**	**95% CI**	***p*-value**	**β**	**95% CI**	***p*-value**	**β**	**95% CI**	***p*-value**	**β**	**95% CI**	***p*-value**

(Intercept)		0.487	[0.419 0.555]	*0.001*	0.547	[0.488 0.605]	*0.001*	0.539	[0.461 0.617]	*0.001*	-0.577	[-1.612 0.458]	*0.273*	0.153	[-0.651 0.957]	*0.708*	-0.421	[-1.597 0.754]	*0.478*

GS	LG	**0.085**	[0.010 0.161]	**0.026**	**0.061**	[0.011 0.125]	**0.042**	**0.073**	[0.013 0.151]	**0.046**	1.053	[0.083 2.195]	**0.047**	-0.155	[-1.033 0.723]	0.728	0.594	[-0.696 1.885]	0.362
	MG	0.025	[-0.049 0.100]	0.499	0.045	[-0.019 0.109]	0.170	0.027	[-0.056 0.111]	0.514	1.555	[0.426	2.684] **0.007**	-0.074	[-0.949 0.800]	0.867	0.928	[-0.352 2.209]	0.153
	SG	-0.031	[-0.116 0.052]	0.458	-0.031	[-0.105 0.042]	0.403	-0.053	[-0.147 0.041]	0.268	**1.995**	[0.676 3.313]	**0.003**	0.682	[-0.332 1.698]	0.187	**1.309**	[0.211 2.899]	**0.036**

PG	LG	-0.114	[-0.189 -0.032]	**0.021**	-0.047	[-0.130 0.034]	0.255	0.002	[-0.113 0.118]	0.971	1.010	[-0.419 2.441]	0.165	0.590	[-0.546 1.728]	0.307	0.546	[-1.188 2.280]	0.534
	MG	0.007	[-0.071 0.087]	0.845	0.016	[-0.051 0.084]	0.640	0.033	[-0.055 0.121]	0.461	1.552	[0.339 2.765]	**0.012**	0.180	[-0.748 1.108]	0.702	0.836	[-0.504 2.177]	0.218
	SG	-0.020	[-0.098 0.057]	0.152	-0.034	[-0.102 0.033]	0.321	-0.055	[-0.143 0.033]	0.219	1.213	[0.026 2.400]	**0.045**	-0.369	[-1.291 0.552]	0.430	0.994	[-0.348 2.337]	0.144

TG	LG	0.026	[-0.056 0.109]	0.525	0.022	[-0.046 0.092]	0.516	0.055	[-0.037 0.148]	0.237	-0.167	[-1.400 1.065]	0.789	-0.802	[-1.738 0.134]	0.093	-0.378	[-1.798 1.040]	0.596

	MG	0a			0a			0a			0a			0a			0a		

ApP = area per player; β = standardized regression coefficient; CI = Confidence interval; GS = game simulation; PG = possession game; TG = tactical game; LG = large game; MG = medium game; SG = small game.

## DISCUSSION

The study aims to develop a similarity and overload score to compare training SSGs and match effort using external load data. Particularly, the similarity score aimed to quantify the ability of SSGs to replicate match kinematic and mechanical demands. Instead, the overload score was established if the SSGs were more demanding or less demanding than the match. The novelty of the study is to develop these two scores to simplify information from multiple GPS variables, to improve data communication and decision-making processes. The second purpose of the study was to analyse the behaviour of the scores in relation to the SSG characteristics in order to understand their usefulness within a real context. The main findings suggest that the similarity and overload scores were significantly higher in the First team compared to the U19 team. Moreover, significant differences were found according to the drills’ format and playing position.

### Development of Similarity and Overload Scores

The principal aim of the current study was to develop a similarity and overload score in order to quantify the ability of SSGs to replicate match intensity. At the end of the process, we had produced three different similarity scores (Sim_kin_, Sim_met_, Sim_mec_) and three overload scores (OVER_kin_, OVER_met_, OVER_mec_) for each SSG and each player. Before moving toward further investigations, a correlation analysis was performed to understand the relationship between the different scores. Sim_kin_ and Sim_met_ (*r* = 0.892, *p* < 0.01), as well as OVER_kin_ and OVER_met_ (*r* = 0.708, *p* < 0.01), showed a significant very large correlation. Even in previous studies [35, 36], kinematic variables (i.e., total distance, high-speed running) showed near perfect correlation with the metabolic load (i.e., high metabolic power). The scores being highly correlated and, therefore, providing similar results, Sim_met_ and OVER_met_ were excluded from further analysis. At the end of the process, the two similarity scores (Sim_kin_, Sim_mec_) and the two overload scores (OVER_kin_, OVER_mec_) were compared in relation to the team, SSG characteristics, and playing roles.

### Similarity and Overload Scores Differences between Teams, SSGs, and Playing Roles

Following calculating similarity and overload scores, the second purpose of the study was to evaluate how values change according to the team (First team vs. U19 team) and according to the SSG characteristics. This last investigation was conducted for the three different roles (i.e., defenders, midfielders, and forwards) identified within the current study. The results suggest that the First team achieved higher similarity and overload scores except for Sim_mec_, where no significant differences were identified (Table 2). To the best of our knowledge, no studies have investigated differences in SSGs between professional and youth soccer players. Houtmeyers et al. [37] only analysed differences in weekly load between U19 and First team players within a professional soccer team. Although the authors considered the overall training sessions, in line with our study, the U19 team registered a shorter distance per minute in low- and high-velocity zones. We could speculate that young soccer players, possessing less technical skills and physical capabilities, are involved in SSGs characterized by lower intensity. As proof of this, Dellal et al. [38] analysed the differences between amateur and professional players. The authors found that amateur players were able to perform less total distance in sprinting and showed lower technical abilities, as highlighted by the higher number of lost balls and a more significant number of skill errors. In addition, Fenner et al. [39] demonstrated that the more talented young players were able to cover a greater distance at higher speeds during SSGs. For this reason, physical and technical capabilities could be crucial elements to ensure SSGs’ intensity, explaining differences between the First team and the U19 team found in the current study. However, we must also consider that different coaching styles and philosophies (e.g., technical and tactical requests) could affect the way of training and, consequently, SSGs’ intensity.

After analysing the differences between the First and U19 teams, our study aimed to understand how similarity and overload scores changed with SSG characteristics. Particularly, we classified SSGs based on the type of drill (game simulations, possession games, tactical games) and size of area per player (small games, medium games, large games). In general, the results suggest that the game simulations allowed the highest similarity scores to be achieved for kinematic and mechanical variables compared with the reference category “tactical games-medium games”.

Game simulations appeared to elicit greater Sim_kin_ in all the playing roles. It is taken for granted that game simulations, due to the presence of goals, could most replicate the match effort. Indeed, in this type of drill, the players’ movement patterns will be more linear as there is a direction to target [17]. In contrast, possession games, characterized by multidirectional movements, induced a significant reduction of the Sim_kin_ score in midfielders, as highlighted by the β coefficient. However, for the game simulations, not all the pitch sizes are able to maximize the Sim_kin_ score. As reported in Table 3, significantly higher scores were found only for medium and large games. In line with previous studies [14, 15, 40, 41], an increase in ApP leads to more space to cover, consequently allowing an increase in game intensity and higher speeds to be reached. In similar populations of both adult [27] and U19 [42] Serie A soccer players, it has been previously demonstrated that a large ApP is required as a tool to replicate official match demands. Indeed, if we consider the β coefficient, large games seem to be the most effective for increasing the Sim_kin_ in the three different playing roles. Regarding the Sim_mec_, only game simulations performed as large games produced a significant increase in the score in the three different playing roles. A greater ApP allows for achieving very high speeds, and consequently, maximum accelerations and decelerations [17]. As in the case of Sim_kin_, possession games (large games) caused a significant reduction in the Sim_mec_ score for defenders.

Examining the results achieved for overload scores, different information was obtained. If the similarity score was developed to understand how the SSGs were able to replicate match intensity, the overload score was integrated to understand how much the SSGs were globally more or less demanding compared to the match intensity. For the OVER_kin_ score, the game simulation – large game combination was identified as the format more suitable for defenders. Interestingly, for midfielders and forwards, the combination possession game – large game was able to maximize the OVER_kin_ score.

Generally, due to their technical abilities, midfielders and forwards are critical players in keeping possession of the ball and applying pressure to win it back. For this reason, when the game aims to maintain possession, midfielders and forwards are more involved, consequently increasing their game intensity. By contrast, for the OVER_mec_ score, the results suggest that by reducing the playing space (game simulation – small game for defenders and forwards), it is possible to have the maximum increase in the overload score. This result confirms previous studies [17] where the total number of changes in velocity (accelerations and decelerations) increased as the pitch size decreased. Consequently, small spaces could be adequate to overload mechanical work intensity compared to the match effort but failed to overload high-speed running and sprints [14, 27, 43]. In contrast, no significant differences were observed for the OVER_mec_ score in the midfielders. As suggested by Riboli et al. [27], the different playing positions need different ApPs according to their specific performance model. Therefore, coaches and physical trainers should be aware that the same SSG could elicit different stimuli for different playing roles [14].

The current study has some limitations. First, the small sample size led to players being grouped in only three different playing positions (i.e., defenders, midfielders, forwards) and there being only a few observations for a specific SSG format (possession game – large game). Moreover, only two teams were included in the current study, and specific aspects such as coaching style and club philosophy could have affected the results. In addition to external load data, internal load parameters (e.g., heart rate) could provide additional comparisons between SSGs and match demands. However, it is necessary to recognize that there could be some technological limitations in constantly monitoring the internal load [16]. Another limitation concerns the SSGs’ characteristics. Indeed, only the type of drill and area per player were considered in the current study. This last point opens up the opportunity for further investigations that could investigate how different SSGs’ characteristics (e.g., number of players, minutes of play, number of sets, recovery time between the sets) affect similarity scores and overload scores.

### Practical applications

**FIG. 3:**
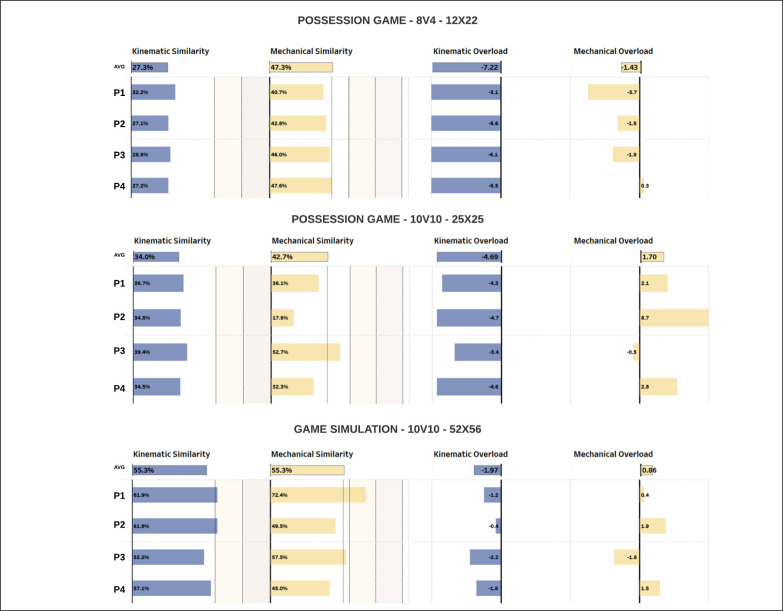
A real example of data visualization and communication of the similarity and overload scores for three different SSGs during a training session. Note: Avg = average value P1 = player 1 P2 = player 2 P3 = player 3 P4 = player 4

The similarity and overload scores developed in this study could be used to classify the different SSGs throughout the season and used in the weekly microcycle in relation to the needs of coaches and physical trainers. Euclidean distance and the average of the pairwise difference between vectors proved to be effective in managing multiple GPS variables, preventing the risk of “data overload”. Indeed, in this way, it is possible to use a single number to understand the general effort achieved during SSGs in relation to match intensity. Although our approach was developed using the WIMU system, it can be replicated with any other GPS system, and also by modifying the variables inserted in the training and match vectors. This method could help the coaching staff in data interpretation and decision-making strategies. To make this process clearer, we created a visualization that could help to understand the utility of these metrics on a daily basis (Figure 3). Particularly, high overload scores could be encouraged at the beginning of the weekly microcycle and away from the match day. On the other hand, high similarity scores should be sought in all the SSGs. Coaches and physical trainers should be aware that game simulations performed in large spaces make it possible to achieve the highest similarity scores for kinematic and mechanical variables. Conversely, game simulations played in smaller spaces could induce an overload in the mechanical parameters. To overload kinematic intensity, possession games should be encouraged. However, it is important to consider that different game formats could lead to different load stimuli.

## CONCLUSIONS

The similarity and overload scores developed in the current study allowed us to compare SSG and match demands. This approach could bring several advantages including the possibility to manage numerous GPS variables, and improve data interpretation and communication. The results suggested that game simulations performed in large spaces made it possible to increase similarity scores for the kinematic and mechanical variables. On the other hand, possession games and smaller play spaces could lead to higher overload scores. Coaches and physical trainers must consider that these results may change according to the level (First team vs U19 team) and role of the players (defenders, midfielders, forwards).

## Supplementary Material

A new approach to comparing the demands of small-sided games and soccer matches
