# Supplementary material for: A new approach to comparing the demands of small-sided games and soccer matches
Source: Biol Sport. 2023 Dec 20;41(3):15–28. doi: 10.5114/biolsport.2024.132989 (PMC11167457; doi:10.5114/biolsport.2024.132989)
Supplement: A new approach to comparing the demands of small-sided games and soccer matches [file JBS-41-51818-s1.pdf]

Supplementary table

S1 TABLE. Small-Sided Games characteristics. Similarity and Overload scores are reported as mean ± SD.

DRILL TYPE	SSG SIZE	PLAYERS' CONTRA-POSITION	PITCH SIZE	PITCH AREA (m^2)	AREA PER PLAYER (m^2)	SIM_{kin}	$OVER_{kin}$	SIM_{mec}	$OVER_{mec}$	N° INDIVIDUAL OBSERVATIONS
Game Simulation	large	4 v 4	50 × 60	3000	375	0.57 ± 0.1	-1.35 ± 1.26	0.61 ± 0.06	-0.81 ± 0.8	14
		5 v 4	50 × 64	3200	356	0.54 ± 0.16	-1.26 ± 2.5	0.59 ± 0.09	-0.56 ± 1.16	14
		6 v 5	52 × 64	3328	303	0.42 ± 0.09	-3.69 ± 1.27	0.46 ± 0.12	0.95 ± 2.61	11
		6 v 6	48 × 52	2496	208	0.63 ± 0.07	-1.04 ± 0.76	0.55 ± 0.16	1.72 ± 1.51	13
		6 v 6	66 × 40	2640	220	0.45 ± 0.14	-3.28 ± 2.92	0.56 ± 0.1	-1.07 ± 0.85	14
		7 v 7	50 × 64	3200	229	0.52 ± 0.16	-2.66 ± 2.82	0.54 ± 0.08	0.92 ± 1.38	13
		7 v 7	52 × 64	3328	238	0.48 ± 0.13	-3.19 ± 2.95	0.56 ± 0.1	0 ± 1.54	36
		7 v 7	55 × 60	3300	236	0.5 ± 0.22	2.93 ± 10.34	0.37 ± 0.16	3.68 ± 2.58	7
		7 v 7	55 × 64	3520	251	0.55 ± 0.1	-1.93 ± 0.91	0.56 ± 0.14	0.76 ± 1.81	14
		7 v 7	60 × 52	3120	223	0.63 ± 0.1	-1.22 ± 0.92	0.61 ± 0.14	0.59 ± 1.29	15
		7 v 7	66 × 50	3300	236	0.45 ± 0.16	-4.63 ± 7.36	0.55 ± 0.09	-1.47 ± 1.09	19
		8 v 7	66 × 64	4224	282	0.54 ± 0.16	-2.38 ± 3.05	0.57 ± 0.1	0.06 ± 1.43	42
		8 v 7	75 × 64	4800	320	0.65 ± 0.16	-1.11 ± 2.48	0.57 ± 0.1	0.57 ± 1.57	14
		8 v 8	52 × 64	3328	208	0.48 ± 0.13	-3.25 ± 2.77	0.56 ± 0.09	0.3 ± 1.33	94
		8 v 8	66 × 60	3960	248	0.61 ± 0.19	0.49 ± 4.21	0.58 ± 0.12	0.81 ± 1.14	12
		8 v 8	66 × 64	4224	264	0.56 ± 0.14	-2.17 ± 2.28	0.58 ± 0.1	0.29 ± 1.33	29
		8 v 8	75 × 60	4500	281	0.45 ± 0.12	-3.51 ± 3.63	0.56 ± 0.1	-0.07 ± 1.45	19
		8 v 8	75 × 64	4800	300	0.61 ± 0.14	-0.97 ± 1.15	0.62 ± 0.08	-0.12 ± 1.12	14
		9 v 8	66 × 64	4224	248	0.6 ± 0.12	-1.7 ± 1.21	0.58 ± 0.08	0.14 ± 1.02	12
		9 v 9	100 × 50	5000	278	0.65 ± 0.11	-0.13 ± 1.16	0.62 ± 0.09	0.39 ± 0.9	14
		9 v 9	66 × 60	3960	220	0.5 ± 0.14	-2.48 ± 2.48	0.56 ± 0.07	-0.54 ± 1.35	14
		9 v 9	66 × 64	4224	235	0.57 ± 0.15	-1.8 ± 1.92	0.58 ± 0.1	0.25 ± 1.55	32
		10 v 10	105 × 60	6300	315	0.58 ± 0.16	3.67 ± 18.27	0.56 ± 0.13	1.07 ± 1.73	40
		10 v 10	105 × 64	6720	336	0.62 ± 0.13	0.39 ± 1.54	0.62 ± 0.1	-0.12 ± 1.29	99
		10 v 10	105 × 68	7140	357	0.54 ± 0.1	1.77 ± 1.07	0.62 ± 0.1	0.53 ± 1.13	19
		10 v 10	66 × 64	4224	211	0.49 ± 0.13	-2.87 ± 2.12	0.57 ± 0.11	-0.81 ± 1.36	67
		10 v 10	66 × 70	4620	231	0.68 ± 0.12	-0.14 ± 4.24	0.65 ± 0.1	0.29 ± 1.13	74
		10 v 10	66 × 78	5148	257	0.65 ± 0.12	-0.72 ± 1.07	0.67 ± 0.08	-0.15 ± 0.87	15
		10 v 10	75 × 60	4500	225	0.54 ± 0.14	-1.97 ± 1.58	0.55 ± 0.1	-0.38 ± 1.73	15

S1 TABLE. Continue.

DRILL TYPE	SSG SIZE	PLAYERS' CONTRA-POSITION	PITCH SIZE	PITCH AREA (m^2)	AREA PER PLAYER (m^2)	SIM_{kin}	$OVER_{kin}$	SIM_{mec}	$OVER_{mec}$	N° INDIVIDUAL OBSERVATIONS
Game Simulation	medium	4v4	32 × 30	960	120	0.37 ± 0.1	-4.85 ± 3.01	0.52 ± 0.07	0.18 ± 1.29	31
		4v4	34 × 30	1020	128	0.36 ± 0.08	-4.55 ± 1.41	0.37 ± 0.09	3.07 ± 1.43	8
		4v4	34 × 40	1360	170	0.33 ± 0.1	-5.64 ± 3.01	0.49 ± 0.1	-0.82 ± 1.69	14
		4v4	50 × 30	1500	188	0.59 ± 0.12	-1.33 ± 1.11	0.53 ± 0.06	1.07 ± 0.74	9
		5v2	33 × 30	990	141	0.44 ± 0.13	-3.81 ± 2.95	0.57 ± 0.09	-0.17 ± 1.42	14
		5v5	35 × 40	1400	140	0.45 ± 0.13	-3.61 ± 2.92	0.48 ± 0.1	0.85 ± 2.05	35
		5v5	35 × 45	1575	158	0.38 ± 0.14	-4.83 ± 3.58	0.46 ± 0.12	-0.82 ± 2.59	12
		5v5	40 × 32	1280	128	0.58 ± 0.11	-1.42 ± 1.18	0.48 ± 0.13	1.7 ± 1.67	45
		5v5	50 × 30	1500	150	0.57 ± 0.11	-1.78 ± 0.8	0.49 ± 0.1	1.86 ± 1.34	11
		5v5	50 × 40	2000	200	0.49 ± 0.16	-2.11 ± 6	0.55 ± 0.11	0.69 ± 2.36	50
		6v4	35 × 50	1750	175	0.4 ± 0.11	-4.04 ± 1.89	0.43 ± 0.1	1.8 ± 2.57	9
		6v5	25 × 50	1250	114	0.4 ± 0.09	-3.67 ± 1.62	0.42 ± 0.11	1.23 ± 2.42	11
		6v6	32 × 40	1280	107	0.37 ± 0.12	-5.31 ± 4.66	0.55 ± 0.1	0.19 ± 1.55	12
		6v6	40 × 32	1280	107	0.51 ± 0.07	-2.29 ± 0.86	0.56 ± 0.1	1 ± 0.98	13
		6v6	45 × 52	2340	195	0.62 ± 0.1	-0.85 ± 1.06	0.51 ± 0.15	1.82 ± 1.6	11
		6v6	50 × 40	2000	167	0.53 ± 0.13	-2.25 ± 1.47	0.51 ± 0.09	1.45 ± 1.3	12
		6v6	52 × 40	2080	173	0.64 ± 0.1	-1.2 ± 0.59	0.61 ± 0.13	1.22 ± 1.15	9
		6v6	53 × 40	2120	177	0.66 ± 0.12	-0.61 ± 0.85	0.56 ± 0.09	0.7 ± 1.12	22
		7v7	38 × 42	1596	114	0.46 ± 0.09	-3.12 ± 1.24	0.47 ± 0.12	1.33 ± 1.14	10
		7v7	40 × 52	2080	149	0.61 ± 0.1	-1.2 ± 0.98	0.56 ± 0.09	1.1 ± 1.06	27
		7v7	50 × 32	1600	114	0.4 ± 0.1	-4.19 ± 2.38	0.57 ± 0.08	-0.36 ± 0.94	13
		7v7	50 × 40	2000	143	0.44 ± 0.13	-3.67 ± 2.43	0.52 ± 0.11	0.29 ± 1.97	13
		7v7	50 × 48	2400	171	0.5 ± 0.11	-2.56 ± 1.61	0.55 ± 0.11	0.71 ± 1.33	22
		7v7	55 × 40	2200	157	0.59 ± 0.1	-1.54 ± 1.05	0.54 ± 0.15	1.16 ± 1.98	13
		7v7	55 × 50	2750	196	0.44 ± 0.11	-3.49 ± 2.29	0.57 ± 0.1	0.2 ± 1.48	27
		7v7	66 × 40	2640	189	0.54 ± 0.14	-2.1 ± 2.67	0.59 ± 0.12	0.69 ± 1.35	27
		8v8	40 × 52	2080	130	0.61 ± 0.17	-1.46 ± 1.10	0.58 ± 0.18	-0.34 ± 2.87	11
		8v8	50 × 52	2600	163	0.55 ± 0.1	-2.05 ± 0.91	0.54 ± 0.1	1.05 ± 1.19	15
		8v8	50 × 60	3000	188	0.45 ± 0.13	-3.62 ± 2.35	0.58 ± 0.09	-0.43 ± 1.09	14
		8v8	54 × 52	2808	176	0.61 ± 0.11	-1.44 ± 0.9	0.56 ± 0.09	0.79 ± 1.43	15
		8v8	66 × 40	2640	165	0.53 ± 0.12	-2.29 ± 1.02	0.57 ± 0.07	0.23 ± 0.91	11
		9v9	50 × 50	2500	139	0.57 ± 0.11	-1.96 ± 1.02	0.54 ± 0.12	0.9 ± 1.9	13
		9v9	52 × 64	3328	185	0.45 ± 0.13	-3.49 ± 2.49	0.55 ± 0.09	-0.16 ± 1.43	32
		9v9	54 × 52	2808	156	0.61 ± 0.09	-1.3 ± 0.83	0.56 ± 0.11	0.99 ± 1.29	13
		9v9	55 × 64	3520	196	0.44 ± 0.11	-3.68 ± 2.69	0.55 ± 0.08	-0.1 ± 1.21	17
		9v9	56 × 53	2968	165	0.58 ± 0.11	-1.74 ± 0.92	0.58 ± 0.13	0.03 ± 1.53	36
		9v9	60 × 52	3120	173	0.64 ± 0.09	-1.11 ± 0.72	0.54 ± 0.13	1.08 ± 1.66	19
		10v10	50 × 60	3000	150	0.47 ± 0.12	-3.29 ± 3.1	0.56 ± 0.1	-0.17 ± 1.52	31
		10v10	52 × 60	3120	156	0.61 ± 0.11	-1.09 ± 1.05	0.58 ± 0.12	0.88 ± 1.24	37
		10v10	52 × 64	3328	166	0.41 ± 0.11	-4.11 ± 3.27	0.55 ± 0.1	-0.49 ± 1.35	33
		10v10	52 × 66	3432	172	0.67 ± 0.12	-0.46 ± 0.89	0.56 ± 0.09	1.21 ± 1.25	19
		10v10	52 × 68	3536	177	0.59 ± 0.19	1.31 ± 10.46	0.59 ± 0.18	1.27 ± 2.68	15
		10v10	53 × 60	3180	159	0.58 ± 0.1	-1.74 ± 0.8	0.58 ± 0.12	0.31 ± 1.56	22
		10v10	55 × 52	2860	143	0.58 ± 0.11	-1.61 ± 1.05	0.6 ± 0.1	0.23 ± 1.29	18
		10v10	56 × 52	2912	146	0.59 ± 0.09	-1.33 ± 0.9	0.57 ± 0.12	0.78 ± 1.52	61
		10v10	56 × 53	2968	148	0.6 ± 0.11	-1.54 ± 0.88	0.55 ± 0.12	0.86 ± 1.58	42
		10v10	60 × 52	3120	156	0.59 ± 0.1	-1.5 ± 0.92	0.57 ± 0.11	0.79 ± 1.39	152
		10v10	60 × 53	3180	159	0.56 ± 0.07	-1.73 ± 0.73	0.54 ± 0.13	1.06 ± 1.47	22
		10v10	60 × 64	3840	192	0.49 ± 0.13	-2.6 ± 2.31	0.48 ± 0.13	1.55 ± 1.62	17
		10v10	62 × 53	3286	164	0.61 ± 0.12	-1.39 ± 0.91	0.53 ± 0.11	1.05 ± 1.61	42
		10v10	64 × 52	3328	166	0.59 ± 0.09	-1.64 ± 0.62	0.6 ± 0.1	0.81 ± 0.8	17
		10v10	66 × 53	3498	175	0.62 ± 0.13	-1.42 ± 1.03	0.58 ± 0.11	0.47 ± 1.39	20
		10v10	66 × 60	3960	198	0.56 ± 0.14	-1.95 ± 2.62	0.6 ± 0.1	-0.11 ± 1.35	78

S1 TABLE. Continue.

DRILL TYPE	SSG SIZE	PLAYERS' CONTRA-POSITION	PITCH SIZE	PITCH AREA (m^2)	AREA PER PLAYER (m^2)	SIM_{kin}	$OVER_{kin}$	SIM_{mec}	$OVER_{mec}$	N° INDIVIDUAL OBSERVATIONS
Game Simulation	small	3 v 3	18 × 15	270	45	0.42 ± 0.08	-3.35 ± 0.83	0.3 ± 0.09	5.18 ± 2.85	12
		4 v 4	32 × 22	704	88	0.37 ± 0.15	-5.54 ± 5.01	0.39 ± 0.08	2.45 ± 1.38	5
		4 v 4	40 × 20	800	100	0.52 ± 0.23	-3.72 ± 5.3	0.4 ± 0.09	2.72 ± 1.82	7
		5 v 5	40 × 20	800	80	0.32 ± 0.11	-6.86 ± 6.52	0.48 ± 0.08	-2.13 ± 1.24	9
		5 v 5	40 × 25	1000	100	0.41 ± 0.07	-3.65 ± 1	0.51 ± 0.06	0.42 ± 0.91	11
		7 v 7	40 × 25	1000	71	0.36 ± 0.07	-4.71 ± 1.18	0.53 ± 0.05	-0.65 ± 1.11	11
		7 v 7	40 × 32	1280	91	0.49 ± 0.09	-2.59 ± 0.96	0.54 ± 0.12	0.89 ± 1.51	44
Possession Game	large	3 v 2	52 × 64	3328	666	0.23 ± 0.09	1.85 ± 5.94	0.38 ± 0.09	0.04 ± 1.91	15
		6 v 4	52 × 64	3328	333	0.39 ± 0.18	-5.41 ± 6.44	0.46 ± 0.09	0.29 ± 2.03	8
Possession Game	medium	2 v 2	30 × 20	600	150	0.3 ± 0.11	-7.09 ± 6.62	0.43 ± 0.11	-2.08 ± 1.95	16
		5 v 5	50 × 40	2000	200	0.5 ± 0.17	-3.31 ± 3.56	0.51 ± 0.06	0.83 ± 1.33	14
		6 v 6	30 × 55	1650	138	0.57 ± 0.14	-0.1 ± 5.39	0.46 ± 0.12	2.11 ± 2.29	20
		6 v 6	35 × 40	1400	117	0.4 ± 0.15	-5.61 ± 8.01	0.47 ± 0.1	1.28 ± 1.87	19
		6 v 6	40 × 32	1280	107	0.51 ± 0.11	-2.53 ± 1.71	0.61 ± 0.1	-0.39 ± 1.29	32
		6 v 6	40 × 35	1400	117	0.49 ± 0.14	-2.77 ± 1.74	0.49 ± 0.11	1.16 ± 1.48	14
		7 v 7	50 × 40	2000	143	0.39 ± 0.13	-4.6 ± 3.52	0.52 ± 0.07	-0.96 ± 1.2	13
		9 v 8	50 × 64	3200	188	0.45 ± 0.07	-3.1 ± 1.03	0.54 ± 0.09	0.12 ± 1.46	12
		9 v 9	50 × 40	2000	111	0.45 ± 0.11	-3.3 ± 2.78	0.55 ± 0.09	0.83 ± 0.83	18
		10 v 10	35 × 60	2100	105	0.42 ± 0.15	-4.71 ± 5.4	0.61 ± 0.11	-0.56 ± 1.07	18
		10 v 10	40 × 60	2400	120	0.48 ± 0.13	-1.96 ± 3.96	0.53 ± 0.13	1 ± 1.76	30
		10 v 10	50 × 50	2500	125	0.45 ± 0.12	-3.04 ± 2.17	0.57 ± 0.08	0.27 ± 0.96	15
		10 v 10	60 × 64	3840	192	0.48 ± 0.13	-2.48 ± 2.29	0.44 ± 0.15	2.21 ± 1.99	17
Possession Game	small	4 v 4	16 × 12	192	24	0.3 ± 0.11	-7.35 ± 7.77	0.47 ± 0.09	-0.2 ± 1.85	33
		4 v 4	16 × 16	256	32	0.26 ± 0.09	-8.43 ± 6.11	0.46 ± 0.11	-0.38 ± 2.04	10
		4 v 4	20 × 18	360	45	0.26 ± 0.1	-10.31 ± 12.58	0.39 ± 0.12	-4.16 ± 2.1	10
		4 v 4	20 × 20	400	50	0.32 ± 0.06	-5.43 ± 1.59	0.41 ± 0.11	1.43 ± 2.73	10
		4 v 4	20 × 22	440	55	0.3 ± 0.09	-6.63 ± 4.97	0.44 ± 0.09	0.66 ± 2.55	19
		4 v 4	25 × 20	500	63	0.28 ± 0.12	-4.82 ± 7.4	0.3 ± 0.12	4.58 ± 4.32	8
		5 v 5	25 × 20	500	50	0.34 ± 0.1	-5.69 ± 3.94	0.46 ± 0.11	0.06 ± 2.24	17
		5 v 5	30 × 20	600	60	0.36 ± 0.1	-5.01 ± 3.51	0.53 ± 0.1	-0.02 ± 1.48	32
		6 v 3	16 × 12	192	21	0.26 ± 0.09	-8.36 ± 5.83	0.5 ± 0.07	-1.71 ± 1.18	14
		6 v 6	20 × 30	600	50	0.41 ± 0.07	-3.47 ± 1.08	0.47 ± 0.14	1.06 ± 2.18	11
		6 v 6	30 × 35	1050	88	0.46 ± 0.09	-3.01 ± 1.11	0.57 ± 0.1	-0.61 ± 1.13	15
		7 v 3	16 × 10	160	16	0.31 ± 0.12	-7.61 ± 9.3	0.48 ± 0.1	-0.95 ± 2.03	18
		7 v 7	33 × 35	1155	83	0.47 ± 0.11	-2.86 ± 1.36	0.43 ± 0.11	1.83 ± 1.88	11
		7 v 7	34 × 20	680	49	0.33 ± 0.09	-5.56 ± 2.57	0.48 ± 0.07	0.18 ± 1.3	16
		7 v 7	40 × 32	1280	91	0.48 ± 0.09	-2.56 ± 1.11	0.46 ± 0.13	1.93 ± 2.25	22
		8 v 4	20 × 15	300	25	0.3 ± 0.09	-6.95 ± 5.21	0.5 ± 0.11	0.37 ± 1.68	12
		8 v 8	30 × 25	750	47	0.36 ± 0.11	-5.11 ± 3.42	0.52 ± 0.06	-0.95 ± 1.06	13
		9 v 9	32 × 40	1280	71	0.38 ± 0.08	-4.37 ± 1.75	0.53 ± 0.1	0.38 ± 0.99	17
		10 v 5	30 × 30	900	60	0.36 ± 0.1	-5.13 ± 3.67	0.48 ± 0.12	1.09 ± 1.83	15
		10 v 10	50 × 40	2000	100	0.44 ± 0.12	-3.46 ± 1.92	0.56 ± 0.07	0.49 ± 1.29	15

S1 TABLE. Continue.

DRILL TYPE	SSG SIZE	PLAYERS' CONTRA-POSITION	PITCH SIZE	PITCH AREA (m^2)	AREA PER PLAYER (m^2)	SIM_{kin}	$OVER_{kin}$	SIM_{mec}	$OVER_{mec}$	N° INDIVIDUAL OBSERVATIONS
Tactical Game	large	4 v 2	52 × 64	3328	555	0.33 ± 0.11	-5.22 ± 3.99	0.38 ± 0.12	-0.61 ± 3.97	16
		6 v 4	52 × 64	3328	333	0.52 ± 0.12	-2.42 ± 1.3	0.52 ± 0.13	1.14 ± 1.88	16
		6 v 4	55 × 64	3520	352	0.45 ± 0.17	-4.26 ± 4.48	0.58 ± 0.1	-0.48 ± 1.2	16
		7 v 7	52 × 64	3328	238	0.4 ± 0.14	-4.77 ± 3.98	0.53 ± 0.1	-1.07 ± 1.51	41
		7 v 7	75 × 60	4500	321	0.4 ± 0.11	-4.03 ± 2.37	0.5 ± 0.09	-2.11 ± 0.93	19
		8 v 4	66 × 64	4224	352	0.47 ± 0.13	-3.07 ± 1.64	0.56 ± 0.12	-0.46 ± 1.52	16
		8 v 5	52 × 64	3328	256	0.35 ± 0.1	-5.67 ± 4.98	0.5 ± 0.12	-0.23 ± 1.63	16
		8 v 6	66 × 64	4224	302	0.58 ± 0.09	-1.54 ± 0.92	0.56 ± 0.14	1.07 ± 1.39	12
		8 v 8	52 × 64	3328	208	0.4 ± 0.12	-4.42 ± 3.61	0.51 ± 0.13	-0.47 ± 2.33	58
		8 v 8	66 × 54	3564	223	0.49 ± 0.15	-3.24 ± 3.99	0.53 ± 0.09	-0.64 ± 1.64	14
		8 v 8	66 × 60	3960	248	0.39 ± 0.11	-4.32 ± 2.83	0.53 ± 0.1	-1.32 ± 1.08	14
		9 v 9	105 × 64	6720	373	0.6 ± 0.19	-0.38 ± 3.25	0.63 ± 0.09	-0.29 ± 0.97	16
		10 v 8	66 × 64	4224	235	0.47 ± 0.15	-3.4 ± 2.85	0.57 ± 0.12	-1.17 ± 1.34	17
		10 v 9	75 × 60	4500	237	0.59 ± 0.13	-0.75 ± 1.52	0.59 ± 0.1	-0.87 ± 1.15	18
		10 v 9	75 × 64	4800	253	0.49 ± 0.14	-3.23 ± 3.51	0.56 ± 0.1	-0.91 ± 1.35	35
		10 v 10	105 × 64	6720	336	0.41 ± 0.17	-4.96 ± 8.41	0.52 ± 0.1	-1.41 ± 1.6	53
		10 v 10	66 × 64	4224	211	0.5 ± 0.14	-1.54 ± 3.48	0.58 ± 0.1	0.46 ± 1.31	18
		10 v 10	75 × 60	4500	225	0.54 ± 0.17	-2.72 ± 3.32	0.59 ± 0.1	-0.77 ± 1.21	51
		10 v 10	75 × 64	4800	240	0.53 ± 0.11	-2.19 ± 1.4	0.62 ± 0.11	-0.49 ± 1.11	16
Tactical Game	medium	6 v 6	40 × 40	1600	133	0.32 ± 0.12	-7.03 ± 6.88	0.46 ± 0.1	-0.57 ± 2.25	12
		7 v 7	50 × 48	2400	171	0.43 ± 0.11	-3.54 ± 1.55	0.54 ± 0.08	-0.55 ± 1.23	11
		7 v 7	50 × 50	2500	179	0.37 ± 0.09	-4.71 ± 2.13	0.47 ± 0.09	0.89 ± 1.11	13
		9 v 8	52 × 64	3328	196	0.49 ± 0.16	-3.22 ± 3.21	0.58 ± 0.09	-0.81 ± 1.1	17

SIM_{kin} = kinematic similarity score; $OVER_{kin}$ = kinematic overload score; SIM_{mec} = mechanicaìl similarity score; $OVER_{mec}$ = mechanical overload score.
